# Elucidation of the XX/XY Sex Determination System and Development of a Sex-Linked Molecular Marker in the Freshwater Snail *Bellamya purificata*

**DOI:** 10.3390/ani16060916

**Published:** 2026-03-14

**Authors:** Yajun Gao, Yanhong Wen, Shaokui Yi, Yong Lin, Jinxia Peng, Xianhui Pan, Xiaoyun Zhou

**Affiliations:** 1 College of Fisheries, Huazhong Agricultural University, Wuhan 430070, China; gaopanyi116@outlook.com; 2 Liuzhou Aquaculture Technology Extending Station, Liuzhou 545006, China; 3 School of Life Sciences, Huzhou University, Huzhou 313000, China; 4 Guangxi Key Laboratory of Aquaculture Genetics and Breeding, Guangxi Academy of Fishery Sciences, Nanning 530021, China

**Keywords:** *Bellamya purificata*, sex-linked molecular marker, GWAS analysis, transcriptome profiling, sex differentiation

## Abstract

The freshwater snail *Bellamya purificata* is both ecologically and economically significant, exhibiting notable sexual dimorphism in growth and nutritional traits that underscore the importance of breeding of monosex stocks. However, the genetic basis of sex determination remains unclear. Herein, genome-wide association studies (GWASs) combined with transcriptomic analysis were conducted to identify sex-linked markers and candidate genes for this species. GWAS generated 571 significantly sex-associated SNPs and 1853 InDels, corresponding to 44 candidate genes. Multiple significant SNP peaks were detected on chromosomes 1 and 2, with *mrc2* and *mis18bp1* as key candidate genes. A sex-linked InDel marker located within *mis18bp1* can distinguish males and females cost-effectively. Genotype analysis of the sex-associated loci revealed that most females were homozygous while males were heterozygous, suggesting that *B. purificata* has a primarily XX/XY sex determination system. Comparative gonadal transcriptome analyses identified 2996 female-biased and 4281 male-biased genes. Among them, *sry*, *sox8*, *dmrt1* and *dmrt2* may be critical in male sex differentiation, while *β-catenin*, *foxl2*, *esr1* and *nr5a2* may be important in female sex differentiation. Integration of GWAS and transcriptomic data highlighted four pronounced sex-associated candidate genes, including *mis18bp1*, *rnf216*, *tbx1* and *mrc2*. These results provide a valuable foundation for elucidating the genetic mechanisms underlying sex determination and for the development of monosex stocks in *B. purificata*.

## Simple Summary

*Bellamya purificata*, a homotypic synonym of *Sinotaia purificata*, is an ecologically and economically important freshwater snail in China. Elucidation of sex determination mechanism and identification of sex-linked molecular markers are of great importance for breeding of monosex stocks. In this study, we used whole-genome resequencing data and gonadal transcriptome data to develop a sex-linked marker and elucidate the genetic architecture underlying sex determination for this species. Based on the data analysis, we obtained several important sex-associated single-nucleotide polymorphisms (SNPs), insertion–deletions (InDels) and candidate genes. The sex determination system of *B. purificata* follows a primarily XX/XY pattern, characterized by females being homozygous and males being heterozygous. Moreover, a sex-specific marker was successfully developed, which can identify male and female individuals cost-effectively. Our research findings provide a molecular marker for developing monosex stocks and contribute to understanding the genetic basis of sex determination in *B. purificata*.

## 1. Introduction

The freshwater snail *Bellamya purificata* (Gastropoda: Viviparidae), an indigenous species in China, has a broad distribution across ponds, lakes, reservoirs, rivers, and other types of aquatic ecosystems [1]. This snail exhibits a propensity for silty substrates and sustains itself by feeding on organic detritus and algae within its habitat [2,3]. Several studies have demonstrated that freshwater snails play pivotal roles in mitigating eutrophication-driven *Microcystis* blooms in freshwater systems through the consumption of *Microcystis* pellets [4,5]. Consequently, *B. purificata* has been acknowledged as a crucial bioremediation organism and is extensively utilized in China’s freshwater polyculture system to optimize resource exploitation and regulate the cultivation environment [6,7,8].

In addition to its significant ecosystem functions, *B. purificata* is also an economically important edible snail in China. Production of “Liuzhou Luosifen”, a renowned delicacy in Liuzhou, has experienced significant growth, generating over 60 billion yuan in annual economic revenue. *Bellamyinae* snails serve as the exclusive ingredient for this dish, with an annual demand in the Luosifen industry reaching approximately 1.5 million tons [9]. However, the current annual production of cultured freshwater snails in China stands at a mere 94,681 tons as of 2023 [10], indicating a substantial supply–demand disparity. With the Luosifen industry’s burgeoning demand, *Bellamyinae* snails have become important aquaculture species in China. Regrettably, the majority of snails used for cultivation are procured from wild populations, which exhibit slow growth rates and low meat yields [9]. Therefore, the application of genomic resources to breed improved varieties is essential for bolstering productivity and economic value in snail aquaculture.

Like most aquatic animals, numerous gastropod snails exhibit sexual dimorphism, displaying differences in growth rate, maturity age, body size, and/or color pattern between the sexes [11]. For example, female freshwater snails of the genus *Viviparus* are larger than the males, with the former also having more globose shape, wider apertures, and body whorls [12]. Similar sex-related differences in size and shape have been observed in *B. purificata*, in which females are larger and have broader body whorls than males [13]. Furthermore, recent study on *B. purificata* has observed nutritional constituents and flavor substances differences between the sexes, with females possessing higher crude protein, glycogen, and flavor amino acids [14]. Therefore, the development of monosex stocks in *B. purificata* is a necessary breeding strategy that could substantially enhance production efficiency and economic benefits, especially for monosex culture [15]. To reach such aims, it is essential to thoroughly comprehend the genetic architecture underlying sex determination, as well as to identify credible sex-associated molecular markers [16]. However, as a newly emerging aquaculture species, limited genomic resources are available for this snail. In particular, the lack of a comprehensive understanding of the genetic mechanisms underlying sex determination hinders the development of efficient sex-associated molecular markers and subsequently the breeding of monosex stocks in *B. purificata*.

Conventional approaches for developing sex-linked markers, including Restriction Fragment Length Polymorphism (RFLP), Amplified Fragment Length Polymorphism (AFLP), Random Amplified Polymorphic DNA (RAPD), and Simple Sequence Repeat (SSR), have played a pivotal role in identifying such markers across more than 20 aquatic species [17,18]. Despite this, these traditional strategies are currently considered time-consuming, labor-extensive, and relatively inefficient when compared with state-of-the-art genomic technologies. In recent years, next-generation sequencing (NGS)-based genetic studies have offered novel perspectives on the sex determination systems in invertebrate species [19]. Particularly, genome-wide association study (GWAS)-based screening of SNP/InDel markers has become a more efficient, precise and sustainable practical tool for analyzing the genetic mechanisms underlying sex determination, with sex-associated markers and genes having been identified in various aquatic species, such as Pacific abalone (*Haliotis discus hannai* Ino) [20], sea urchin (*Mesocentrotus nudus*) [19], deep-sea mussel (*Gigantidas platifrons*) [21], Portuguese oyster (*Magallana angulata*) [16] and ivory shell (*Babylonia areolate*) [22]. Furthermore, RNA-Seq enables the identification and characterization of genes, along with the accurate quantification of transcript abundance [23]. Additionally, it serves as a valuable tool for deciphering gene regulation networks via the detection of differentially expressed genes (DEGs) [24]. Accumulating evidence indicates that combining GWAS and RNA-Seq can narrow the range of quantitative trait loci (QTLs) and improve the accuracy of identifying candidate genes [25]. This integrative method has been widely adopted in several aquaculture species, such as the sea urchin *Mesocentrotus nudus* [19] and the Japanese grenadier anchovy *Coilia nasus* [25], and has greatly advanced the process of molecular marker-assisted monosex breeding.

In the present study, genome-wide association studies (GWASs) and comparative transcriptome profiling were employed to identify sex-associated loci and elucidate the genetic architecture underlying sex determination in *B. purificata*. The results establish a fundamental framework for investigating the genetic mechanisms of sex differentiation and may facilitate the further development of monosex stocks for this species.

## 2. Materials and Methods

### 2.1. Ethics Statement

All experimental procedures related to snails were performed in strict compliance with the guidelines in the Guide for the Care and Use of Laboratory Animals of Huazhong Agricultural University. All efforts were made to minimize suffering of the snails.

### 2.2. Sample Collection

Five populations were sampled for this study. Among them, two populations consisting of 287 adult individuals (72♂ and 215♀) collected from the rice–crayfish coculture field of Qianjiang (Hubei Province) and Chaohu Lake (Anhui Province) were used for GWAS analysis. Nine males and nine females from the Qianjing population were selected for RNA-Seq. Three populations including those from Honghu Lake (Hubei Province) (53♂ and 97♀), rice–crayfish coculture fields of Tianmen (Hubei Province) (66♂ and 72♀), and Weishan Lake (Shandong Province) (32♂ and 65♀) were collected for sex-linked marker validation. Sampling information was listed in Table S1. Taxonomic identification of the snails was conducted based on morphological characteristics, following the descriptive criteria established by Zhang and Liu [26].

Prior to handling, snails were drained to remove excess water from the mantle cavity and then blotted dry with absorbent paper [27]. Shell width (SW) and total weight (T_W_) were measured to the nearest 0.01 mm and 0.01 g, respectively. Subsequently, the snails were deshelled and individuals were sexed. Males were identified by the presence of brownish-yellow testes and females by the presence of a red protein gland and uterus [26]. Afterwards, the abdominal foot muscle of each individual was separated and preserved in 95% ethanol and stored at −20 °C for DNA extraction. The gonads of adult *B. purificata* were separated and split into two parts; one was fixed with Bouin’s solution for histology to identify phenotypic sex, while the other one was immediately flash-frozen in liquid nitrogen and stored at −80 °C for subsequent RNA extraction.

For histological analysis, Bouin’s solution-fixed gonadal tissues were dehydrated in a graded ethanol series (70–100%), embedded in paraffin, and cut into 5 μm thick sections, followed by hematoxylin–eosin (H&E) staining and observation under a light microscope (4× and 40×).

### 2.3. DNA Extraction and Whole-Genome Resequencing

Genomic DNA of each abdominal foot muscle was isolated using the ammonium acetate method [28]. DNA quality was assessed by 1% agarose gel electrophoresis, while DNA concentration was determined using a Nanodrop 2000 spectrophotometer (Thermo Scientific, Wilmington, DE, USA). Subsequently, the qualified DNA samples were sent to Shanghai Majorbio Bio-pharm Technology Co., Ltd. (Shanghai, China) for DNA library construction, and sequencing was performed on the DNBSEQ-T7 platform (Complete Genomics, a subsidiary of MGI Tech, Shenzhen, China). Raw sequencing reads were subjected to preliminary quality control using fastp (v0.21.0) [29] to eliminate low-quality reads, adapter sequences, and ambiguous bases. Then clean reads were aligned to the reference genome of *B. purificata* (CNGBdb: CNA0142815) using BWA-MEM2 (v2.2.1) [30]. Variant detection and genotyping were performed with the Genome Analysis Toolkit (GATK, v4.4.0.0) [31]. SNPs and InDels were identified using the “HaplotypeCaller” module of GATK. Furthermore, variant filtering was conducted using GATK to eliminate low-quality variants, including those exhibiting elevated strand bias, insufficient mapping quality, aberrant read position, or substandard mapping quality rank sum scores. For SNPs, the filtering thresholds were set as follows: QD < 2.0 || FS > 60.0 || MQ < 40.0 || SOR > 3.0 || ReadPosRankSum < −8.0 || MQRankSum < −12.5. For InDels, the parameters used were QD < 2.0 || FS > 200.0 || SOR > 10.0 || ReadPosRankSum < −20.0 || MQRankSum < −12.5. Additional filtering was performed with VCFtools (v0.1.17) [32], using the following criteria: min-alleles, 2; maf, 0.05; min-meanDP, 3; and max-missing, 0.75. Functional annotation of the identified SNPs and InDels was subsequently carried out using SnpEff v4.3t [33], according to the gene annotation of the reference genome.

### 2.4. Population Genetic Structure and Linkage Disequilibrium Analysis

Before performing genetic association analyses, principal component analysis (PCA) was implemented using PLINK v1.9 [34] based on the filtered genotype data, while population structure was inferred using ADMIXTURE v 1.3.0 [35]. The *K*-value range was set from 1 to 8 during the analysis, and the optimal *K*-value was determined according to the minimum cross-validation error. Phylogenetic trees were constructed using the Maximum Likelihood (ML) method with IQ-TREE2 [36] and the Neighbor-Joining (NJ) approach with FastTree v2.1.11 [37], respectively. Pairwise linkage disequilibrium (LD) and the squared correlation coefficients (*r*^2^) between alleles at each locus were calculated with PopLDdecay v3.41 [38], and the corresponding LD decay curve was constructed.

### 2.5. Sex-Associated Regions and Loci Screening Using GWAS

Based on the filtered SNPs and InDels, genome-wide association studies (GWASs) between sex phenotype and genotype were conducted in rMVP v3.6.0 [39] with three models: the general linear model (GLM) [40], the mixed linear model (MLM) [41], and fixed and random model circulating probability unification (FarmCPU) [42].

### 2.6. Single-Marker and Haplotype Association Analysis

LDBlockShow v1.40 [43] was employed to produce locus-zoom plots and linkage disequilibrium (LD) heatmaps covering the 5.844 kb regions upstream and downstream of the lead SNP in the significant association signals, with the aim of identifying SNPs in strong LD with the lead SNP. Single-marker association analysis was subsequently conducted for SNPs located within the coding sequence (CDS) of genes harboring a high density of genetic markers with strong linkage disequilibrium. In addition, phenotypic validation of InDels in strong LD with significant SNPs was conducted to identify sex-specific fragments distinguishable by gel electrophoresis.

### 2.7. Development and Validation of Sex-Specific Markers

InDels longer than 8 bp were selected for further development of sex-specific markers. Based on the positions of these markers, primers were designed with NCBI Primer-BLAST (https://www.ncbi.nlm.nih.gov/tools/primer-blast/, accessed on 20 March 2025) using the corresponding genomic sequences. The PCR reaction mixture consisted of 12.5 μL of 2 × Taq PCR Mix, 1.0 μL of template DNA, 1.0 μL of both upstream and downstream primers (10 μM), and nuclease-free water to a final volume of 25 μL. PCR reactions were carried out in an ABI Veriti^®^ 96-well Thermal Cycler (Applied Biosystems, Foster City, CA, USA) with the following conditions: initial denaturation at 95 °C for 3 min; 35 cycles of 95 °C for 25 s, 55 °C for 25 s, and 72 °C for 30 s; and 72 °C for 5 min. The amplified PCR fragments were visualized via 3% agarose gel electrophoresis.

### 2.8. RNA Extraction and Transcriptome Analysis

Following sex identification, nine female and nine male gonads were selected for RNA extraction. RNA integrity was assessed using an Agilent 2100 Bioanalyzer (Agilent Technologies, Santa Clara, CA, USA), while RNA concentration was determined with a NanoDrop 2000 spectrophotometer (Thermo Scientific, USA). Qualified RNA was then used for RNA-sequencing analysis. Equal amounts of RNA from three female gonads were combined to generate three RNA pools, and the same procedure was applied to the nine male gonads. Herein, three RNA pools were prepared from both female and male gonads for RNA-Seq analysis. These samples were then sent to Shanghai Majorbio Bio-pharm Technology Co., Ltd. (Shanghai, China) for cDNA library construction. Sequencing of the libraries was performed on the Illumina NovaSeq platform (Illumina, San Diego, CA, USA), generating 150 bp paired-end reads.

Raw sequencing reads were processed and filtered using Trimmomatic (v0.39) (http://www.usadellab.org/cms/?page=trimmomatic, accessed on 26 January 2025) [44] to generate clean reads. These clean reads were aligned to the *B. purificata* reference genome (CNGBdb: CNA0142815) using HISAT2 v2.1.0 [45]. RSEM v1.3.3 [46] was applied to count the assembled transcripts and conduct subsequent normalization for the calculation of RPKM values. DESeq2 v1.40.1 [47] was used to perform differential expression analysis, so as to screen out differentially expressed genes (DEGs) between ovarian and testicular samples with the default threshold of |log_2_ FC| > 1 and *p* < 0.05. Furthermore, Gene Ontology (GO) enrichment analysis and Kyoto Encyclopedia of Genes and Genomes (KEGG) pathway analysis were carried out for these DEGs via DAVID (https://davidbioinformatics.nih.gov/) [48].

To validate the RNA-Seq data, 18 DEGs associated with sex differentiation and gonadal development were selected for qRT-PCR experiment. Primers (Table S2) were designed using Primer Premier 6.0 (https://www.premierbiosoft.com/primerdesign/index.html, accessed on 26 January 2025) based on the gene sequences from the transcriptome data and prepared by Wuhan Tianyi Huayu Gene Technology Co., Ltd. (Wuhan, China). The qRT-PCR was performed on a CFX96TM Real-Time PCR Detection System (Bio-Rad, Hercules, CA, USA). Each qRT-PCR reaction system consisted of 4 μL of forward and reverse primers, 2 μL of cDNA template, 10 μL of 2 × SYBR mix (TaKaRa, Dalian, China), and 7.2 μL of ddH_2_O. Triplicate reactions were performed for each sample, with the *ef1α* gene serving as the internal reference. The amplification program was set as follows: 95 °C for 10 s and 40 cycles of 95 °C for 5 s, 58–60 °C for 20 s, and 72 °C for 20 s. Relative gene expression levels were quantified using the $2^{-\Delta \Delta Ct}$ method.

### 2.9. Candidate Gene Identification and Functional Annotation

After obtaining the loci associated with sex-related traits, flanking regions of 5.844 kb were extracted using bedtools v2.30.0 [49] as the candidate regions. Genes located within these regions were considered potential candidate genes. Significantly differentially expressed genes (DEGs) associated with sexual differentiation identified from RNA-Seq analysis were also considered as potential candidate genes. To refine the candidate gene set, all predicted genes were compared with the RNA-Seq results, and those overlapping with the transcriptomic dataset were considered as key candidate genes.

## 3. Results

### 3.1. Morphological and Histological Appearance of Sexual Characteristics

**Figure 1:**
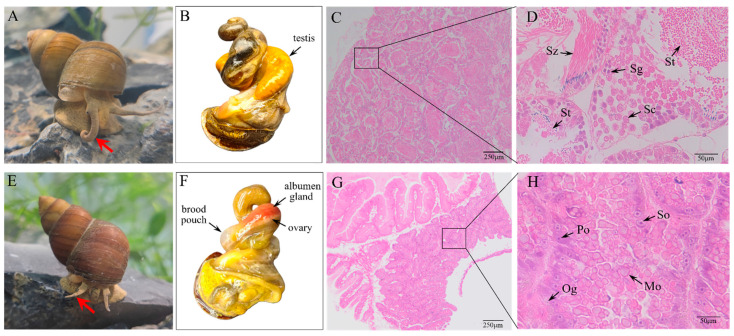
Morphological and histological appearance of sexual characteristics of *B. purificata*. (**A**,**E**) Male and female individuals; the red arrows indicate the differences in right tentacles between males and females. (**B**,**F**) Macrostructure of male and female gonads; (**C**,**D**) testis tissue sections; (**G**,**H**) ovary tissue sections. Scale bars: (**C**,**G**) 250 µm, (**D**,**H**) 50 µm.

*B. purificata* is a dioecious species with a unique ovoviviparous life history. However, male and female individuals can hardly be differentiated morphologically by differences in size or shell color. For individuals with well-extended tentacles, males can be identified by their tightly curved right tentacle, which is thicker and stronger than the left one and functions as the copulatory organ (Figure 1A). The female, on the other hand, has two slender, straight, equal-length tentacles (Figure 1E). After removal of the shell, the mature male can be distinguished by the yellowish testis (Figure 1B), while the female exhibits a pinkish albumen gland and a swollen brood pouch with the eggs and hatching juveniles within it appearing as irregularities on its external surface (Figure 1F). Examination of serial sections of the testis shows the spermatogonia (Sg), spermatocytes (Sc), spermatids (St) and spermatozoa (Sz) distributed within the lumen of the seminiferous tubule (Figure 1C,D), while the ovary contains oogonia (Og), primary oocytes (Po), secondary oocytes (So) and mature oocytes (Mo) (Figure 1G,H).

### 3.2. Genotyping and Population Structure Analyses

Whole-genome resequencing-based genotyping was performed on 215 female and 72 male *B. purificata*, generating a total of 3279.2 Gb of clean data. On average, each sample generated 11.4 Gb of clean data. Relative to the genome size, the average mapping rate reached 98.6%, with a mean sequencing depth of 11.6×.

**Figure 2:**
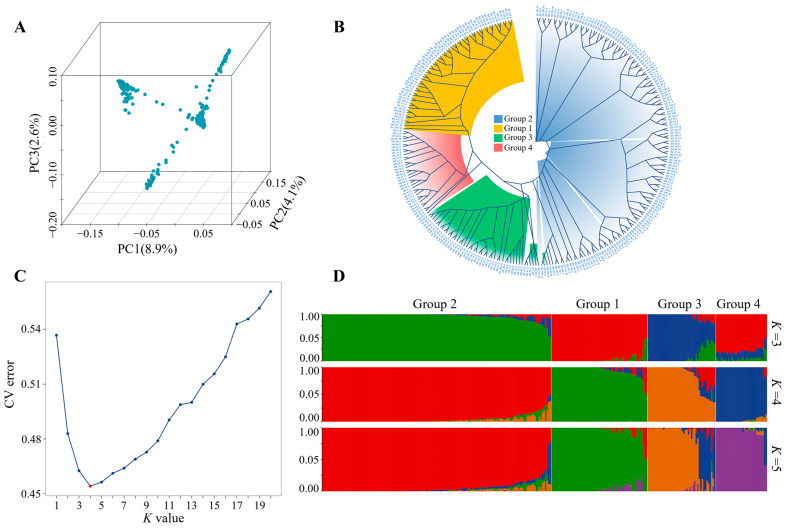
Population structure analysis of the *B. purificata* samples. (**A**) Population structure of *B. purificata* revealed by 3D PCA. (**B**) The NJ phylogenetic tree of the analyzed samples constructed from genotype data. (**C**) Cross-validation error rates corresponding to *K* values from 1 to 20, where *K* denotes the number of population subgroups. (**D**) Population structure analysis results for *K* = 3, 4, and 5. Each column (vertical bar) represents an individual sample. Each color corresponds to an inferred ancestral subpopulation. The proportion of each color indicates the fraction of the individual’s genome derived from the corresponding ancestral subpopulation. The presence of multiple colors within a single vertical bar indicates genetic admixture, i.e., the individual has inherited genetic components from multiple ancestral subpopulations.

Subsequently, we assessed the population structure and genetic relationships among *B. purificata* individuals using principal component analysis (PCA), kinship analysis, and population structure analysis in combination. Both the PCA results (Figure 2A) and the NJ-based phylogenetic tree (Figure 2B) revealed clear population stratification, with 287 individuals forming four distinct genetic clusters. During population structure analysis, the cross-validation error gradually decreased as the *K* value increased, with the lowest value observed at *K* = 4, indicating that the population could be best clustered into four subgroups (Figure 2C,D). These results indicated the presence of significant population structure within the sampled population. To control for the confounding effects of population stratification in the subsequent GWAS, the top three principal components (PC1, PC2, and PC3) with eigenvalues > 1 were incorporated as covariates in the GWAS statistical model.

### 3.3. Variant Calling

**Figure 3:**
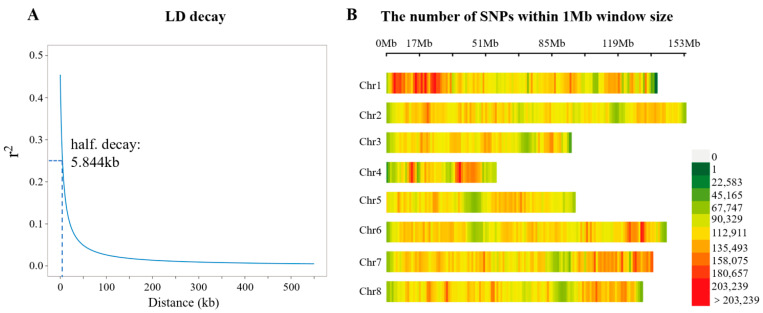
Genetic background information of the analyzed *B. purificata* samples. (**A**) Genome-wide LD decay plots of the study individuals; the X-axis represents physical distance, and the Y-axis represents LD value (*r*^2^). (**B**) Genome-wide identification and distribution analysis of high-quality SNPs on 8 chromosomes of *B. purificata*. The gradient color scale from green to red reflects the increasing SNP density within 1 Mb intervals.

Following stringent quality control procedures, 14,024,434 high-quality SNPs and 2,904,311 InDels were identified across 287 individuals and subsequently used for GWAS. Linkage disequilibrium (LD) decayed rapidly within the population, reaching half of its maximum value (*r*^2^ = 0.239) at an average inter-SNP distance of 5.844 kb (Figure 3A), indicating abundant genetic diversity existed in the analyzed population. The reference genome applied in the present study was approximately 939.19 Mb in length. Theoretically, to guarantee the reliability of association analysis, at least 160,710 evenly distributed genetic markers (939.19 Mb/5.84 kb) would be needed. In this study, the actual number of markers employed (16,928,745) was vastly higher than this threshold. The distribution of SNPs was visualized using a SNP density plot, which revealed a relatively uniform pattern across the eight chromosomes with an average density of one SNP per 352 bp (Figure 3B).

### 3.4. Genome-Wide Association Studies

**Figure 4:**
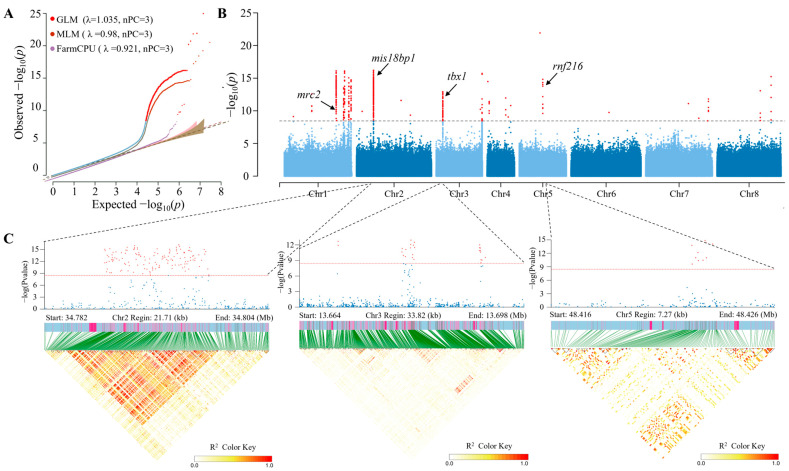
GWAS results for sex traits in *B. purificata*. (**A**) QQ plot. (**B**) Manhattan plot. The X-axis represents chromosome numbers, and the Y-axis shows –log_10_(*p*) values of SNPs. Red dots indicate SNP clusters exceeding the significance threshold. (**C**) Locus-specific zoom plots of the genomic regions surrounding the key associated SNPs.

To identify the optimal model for population stratification correction, three distinct analytical approaches were adopted, the GLM, the MLM, and FarmCPU, all integrated with three principal components (PCs). The corresponding λ values were 1.035, 0.980, and 0.921, respectively. Generally, the optimal λ value ranges from 0.95 to 1.05, with values closer to 1 providing better control of false positives caused by population structure and kinship. Both the MLM (λ = 0.980) and GLM (λ = 1.035) models were suitable for GWAS. However, the GLM generated Manhattan plots consistent with those of the MLM while identifying more candidate SNPs. To retain an adequate number of candidate SNPs, the GLM was chosen for subsequent analysis (Figure 4A). The Manhattan plot revealed that 571 SNPs (Figure 4B, Table S3) and 1853 InDels (Table S4) reached the suggestive threshold for association with sex differentiation and gonadal development processes. Among the 571 sex-specific SNPs, approximately 40.98% were located on chromosome 1 and formed multiple significant SNP peaks. In addition, 30.65% and 20.14% of the SNPs were distributed on chromosomes 2 and 3, respectively, with one significant SNP peak for each chromosome (Figure 4B).

The genotypes of the sex-associated SNPs are summarized in Table S5. Analysis of the homozygous and heterozygous genotypes of the 163,877 SNPs from 287 individuals of 571 loci revealed that 81.01% of male genotypes were heterozygous, while 88.63% of female genotypes were homozygous, which suggests that *B. purificata* likely has a primarily XX/XY sex determination system.

**Figure 5:**
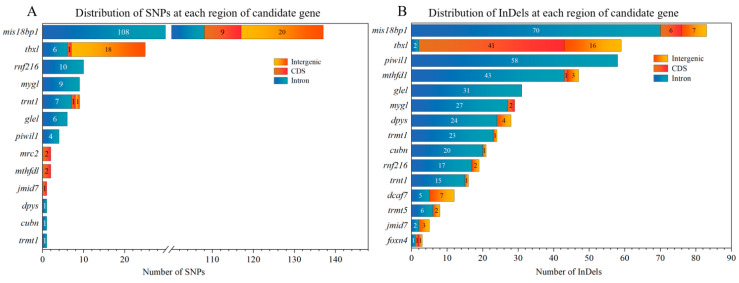
Distribution of SNPs (**A**) and InDels (**B**) within different regions of the respective candidate genes.

We performed functional annotation for these sex-specific SNPs and InDels using SnpEff [33]. It revealed that the sex-associated loci corresponded to 44 candidate genes within linkage disequilibrium regions (Table S6). Furthermore, of the 571 SNPs, 32.75% (187 SNPs) were mapped to six genes, including mis18-binding protein 1 (*mis18bp1*) (137 SNPs), T-box transcription factor (*tbx1*) (25 SNPs), E3 ubiquitin-protein ligase RNF216 (*rnf216*) (10 SNPs), melanocyte proliferating gene 1 (*myg1*) (9 SNPs), piwi-like protein 1 (*piwil1*) (4 SNPs) and C-type mannose receptor 2 (*mrc2*) (2 SNPs) (Figure 5A). Similarly, 13.38% of the 1853 InDels were located at *mis18bp1*, *tbx1*, *rnf216*, *myg1* and *piwil1* (Figure 5B). LocusZoom plots illustrating the linkage disequilibrium between the lead SNP in the *mis18bp1* region (Chr.2-34795421) and all genetic markers within the 5.844 kb flanking region revealed that all SNPs strongly linked to Chr.2-34795421 were concentrated near the *mis18bp1* gene (Figure 4C).

### 3.5. Development and Detection of Sex-Linked Molecular Marker

**Figure 6:**
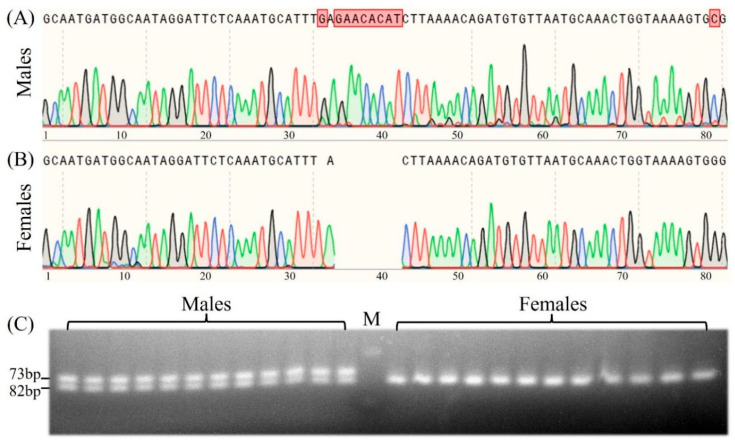
The sex-linked molecular marker of *B. purificata*. (**A**,**B**) Sanger sequencing alignment between males and females across sex-linked loci; (**C**) representative gel electrophoresis profiles obtained from female and male individuals using the primer pairs.

To develop a PCR-based sex-specific molecular marker for *B. purificata*, sixty sexually dimorphic InDels were screened out, and primers were designed according to the genome sequence. After preliminary PCR screening, we successfully obtained an InDel located within the intronic regions of *mis18bp1* gene which effectively distinguished male and female *B. purificata*. Using the locus-specific primer pair (F: 5′-GCAATGATGGCAATAGGATTCTCAA-3′ and R: 5′-CGCACTTTTACCAGTTTGCAT-3′), PCR amplification yielded different-length products, and subsequent Sanger sequencing of the PCR products revealed a female-specific 9 bp deletion and a sex-specific nucleotide site (Figure 6A,B). Following the refinement of electrophoresis conditions (3% agarose gel electrophoresis for 1h), distinct electrophoretic patterns were observed: two bands in males and only one band in females (Figure 6C).

Further validation showed that this locus-specific primer pair could successfully distinguish female and male samples from Honghu Lake, rice–crayfish coculture field of Tianmen, and Weishan Lake (Table S1), indicating the wide applicability of the sex-linked InDel marker. In addition, males exhibiting a heterozygous binding phenotype and females a homozygous binding phenotype at the sex-linked InDel marker suggest that the sex determination system of *B. purificata* is primarily male heterogametic (XX/XY).

### 3.6. Transcriptome Analysis of DEGs Between Testes and Ovaries

**Figure 7:**
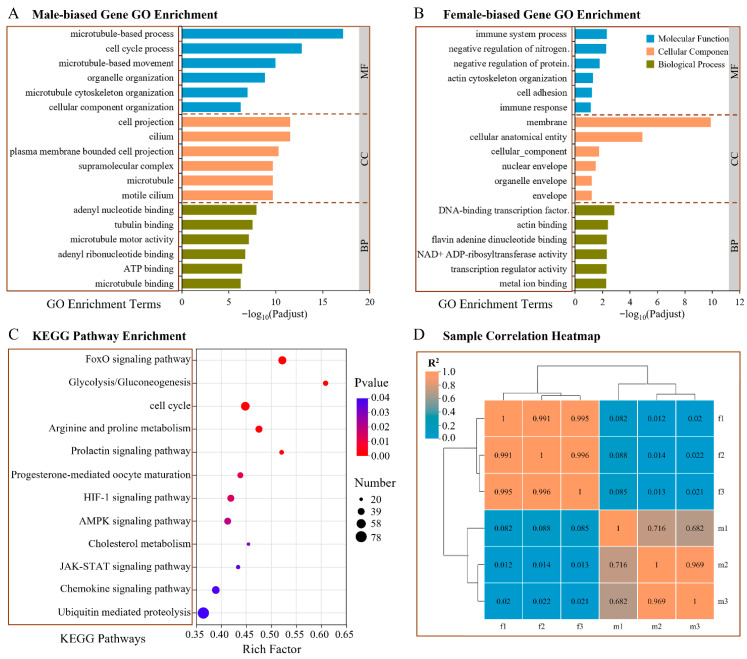
GO (**A**,**B**) and KEGG (**C**) pathway enrichment analyses of DEGs between female and male gonads of *B. purificata*. (**D**) Sample correlation heatmap plot showing the strong clustering associated with sex. The number in each column and row corresponds to the correlation coefficient of one sample with other five samples.

To gain a comprehensive understanding of gonadal transcriptional divergence and clarify the genetic mechanism underlying sex determination in *B. purificata*, we constructed and sequenced cDNA libraries from testis and ovary tissues. DEGs analysis identified a total of 7277 sex-biased genes between testes and ovaries (Figure 7A). Among these, 2996 genes were upregulated in ovaries (female-biased), while 4281 genes showed elevated expression in testes (male-biased) (Table S7). To investigate the potential roles of these DEGs in sex determination, differentiation, and gametogenesis, we performed GO and KEGG pathway enrichment analyses. GO functional enrichment analysis revealed that female-biased genes were primarily enriched in cellular component terms, including membrane and related cellular component categories. Enriched terms in the biological processes category were primarily immune system process and actin cytoskeleton organization, while the molecular functions primarily included DNA-binding transcription factor activity (Figure 7A). The male-biased genes were primarily involved in biological processes, such as microtubule-based process, cell cycle process and microtubule-based movement. The cellular components primarily included the cilium and microtubule, while the molecular functions primarily included tubulin binding and microtubule motor activity (Figure 7B). These findings imply that microtubule-associated functions play vital roles in testicular development, likely affecting biological processes including spermatogenesis and cell division. In the KEGG pathway analysis, 4014 DEGs were annotated against the KEGG database and assigned to 39 distinct pathways. The top 20 significantly enriched pathways are presented in Figure 7C. Some of the DEGs were enriched in the cell cycle, cholesterol metabolism, and FoxO signaling pathways, which are known to participate in the proliferation and function of gonadal cells, as well as regulate the biosynthesis of sex hormones. The glycolysis/gluconeogenesis signaling pathway plays a pivotal role in energy metabolism. Furthermore, the progesterone-mediated oocyte maturation (POM) and prolactin signaling pathways play crucial roles in ovarian function by modulating oocyte maturation and ovulation.

### 3.7. Key Genes Associated with Sex Determination, Differentiation and Gonadal Development

By analyzing the global gene expression profiles of gonads, we identified 68 candidate genes associated with multiple processes of sex determination, differentiation, and gonadal development (Table 1). Among these candidates, sixteen key genes were predominantly implicated in sex determination and differentiation. Specifically, the expression levels of *sry*, *gata4*, *dmrt1*, *dmrt2*, *sox8*, and *hsd11b2* were significantly higher in testes than in ovaries, while the expression levels of *β-catenin*, *esr1*, *Nr5a2 and foxl2* in ovary were higher than those in testis. Twenty-nine key genes were primarily associated with spermatogenesis, among which *piwil1*, *spata4/6/17/20/24*, *klhl10*, and *tex9/11/36/43/47/49/52/55/264* exhibited significantly higher expression levels in the testis. Moreover, seven genes associated with ovarian development and six genes implicated in steroid biosynthesis and metabolism were identified in ovarian and testicular tissues. Notably, key genes known to regulate sex-determination cascades in mammals, including *sry*, *gata4*, *sox8*, *dmrt1*, and *dmrt2* in males, as well as *β-catenin*, *foxl2*, *esr1*, and *nr5a2* in females, were also detected in *B. purificata*. These results suggest that *B. purificata* and mammals share conserved genetic regulatory networks governing sex determination. The RNA-Seq data were validated by qRT-PCR analysis and the results are shown in Figure S1.

### 3.8. Identification of Overlapping Genes Between GWAS and RNA-Seq

In the GWAS, a candidate interval spanning 5.844 kb upstream and downstream of each significant SNP was defined. A total of 44 genes located within these intervals were extracted and functionally annotated (Table S6). These putative genes were further compared with the differentially expressed genes (DEGs) obtained from gonadal RNA-Seq analysis. Seven overlapping genes were identified between the two datasets, including *mis18bp1*, *rnf216*, *tbx1*, *mrc2*, *cubn*, *sirt4*, and *dyps* (Table 2). These genes were distributed across five different chromosomes and showed no obvious clustering.

## 4. Discussion

Over the past few years, GWAS has become recognized as a powerful tool for developing sex-specific markers, owing to its high genome coverage, capacity to identify large sex-linked fragments, and ability to discover abundant genetic markers [15]. This strategy has been widely applied to the development of sex-specific molecular markers in many aquatic species [15,16,19,20,21,22,50]. In this study, we performed a GWAS for sex-related traits in *B. purificata* and detected 571 SNPs and 1853 InDels that were significantly associated with sex phenotype. Among the sex-associated SNPs and InDels, one sex-linked marker located at *mis18bp1* gene was successfully screened out. Diagnostic consistency reached 100% across all three validation populations, demonstrating that this sex-specific marker enables accurate and efficient sex identification in *B. purificata*. This result provides a powerful tool for researchers and breeders to identify the sex of *B. purificata* more easily and accurately.

Sex-determining genes usually display two typical characteristics: (1) the presence of sex-specific genetic variations, such as SNPs and InDels with marked divergence between males and females; (2) distinct expression patterns between testes and ovaries, indicating their functional involvement in sex determination and differentiation [16]. Such differential features have been confirmed in several well-studied sex determination and differentiation genes, including *dmrt1* in freshwater snail (*Cipangopaludina chinensis*) [51] and Pacific oyster (*Crassostrea gigas*) [52], *foxl2* and *dmrt1l* in Yesso scallop (*Patinopecten yessoensis*) [53], and *dmy* in medaka (*Oryzias latipes*) [54]. Genes identified through the overlap of GWAS and sex-biased expression profiles are generally considered to have higher reliability than candidates derived from a single dataset. Combining GWAS and RNA-Seq analyses, we screened seven candidate genes harboring both sex-associated SNPs/InDels and sexually dimorphic expression patterns: *mis18bp1*, *rnf216*, *tbx1*, *mrc2*, *cubn*, *sirt4*, and *dyps*.

Among the seven candidate genes identified, *tbx1* and *rnf216* appear to participate in the regulatory framework involving testicular differentiation and spermatogenesis. *Tbx1* belongs to the T-box family of transcription factors and plays crucial roles during embryogenesis and organogenesis [55]. Specially, TBX1 was originally identified as a “testis-specific T-box protein”, due to its abundant and tissue-specific expression in adult testes of mice and humans [56,57]. TBX1 was also proposed to participate in the regulation of testicular differentiation in aquatic organisms. In rainbow trout (*Oncorhynchus mykiss*), *tbx1* showed significantly higher expression in the testis during sex differentiation, with distinct expression differences between testicular and ovarian development observed soon after hatching [58]. Further studies have indicated that *tbx1* regulates testis development through interaction with the retinoic acid (RA) signaling pathway in rainbow trout (*Oncorhynchus mykiss*) [55].

Another candidate sex-differentiation gene identified in *B. purificata* is *rnf216*, which encodes an E3 ubiquitin ligase. This gene has been reported to be indispensable for spermatogenesis and male fertility in mammals [59]. In mice, *rnf216* exhibited remarkably high expression in the testis. During postnatal testicular development, *rnf216* transcripts were detectable as early as 1 week postpartum, and its expression level gradually increased from 2 weeks of age through adulthood. *Rnf216*-deficient mice displayed reduced testis size and extensive germ cell depletion. Further evidence indicated that germ cell degeneration in these mice initiated at 2 weeks of age and peaked at 3–4 weeks, corresponding to a critical window for spermatogenesis [59]. Notably, targeted disruption of *rnf216* lead to male-specific infertility, with no significant fertility defects observed in females. Ablation of *rnf216* impairs meiosis prophase I: *rnf216^-/-^* spermatocytes were incompletely arrested at the zygotene stage and subsequently underwent apoptosis at approximately the pachytene stage. Mechanistic investigations further demonstrated that RNF216 sustains normal meiotic progression by mediating the degradation of protein kinase A (PKA) catalytic subunits [60]. Although the function of *rnf216* remains poorly characterized in aquatic organisms, emerging evidence from RNF family members supports their conserved roles in sexual regulation. For instance, *rnf183* was identified as a sex-determining gene in yellow croaker (*Larimichthys polyactis*) [61], and *rnf144a* has been proposed as candidate sex-determining gene in blotched snakehead (*Channa maculata*) [62] and Northern snakehead (*C. argus*) [15].

*Mrc2* (C-type mannose receptor 2) encodes an endocytic collagen receptor that participates in extracellular matrix remodeling by mediating collagen internalization and subsequent lysosomal degradation [63]. In this study, two SNPs were identified within the *mrc2* genomic region, and transcriptome analysis revealed that this gene exhibited markedly higher expression in the ovary than in the testis. The Wnt/*β*-catenin signaling pathway is known to be an evolutionarily conserved and ancient cascade responsible for ovarian determination in diverse vertebrate groups, such as mammals, amphibians, and teleost fishes [64]. According to recent evidence, *mrc2* acts as an upstream regulator of the Wnt/*β*-catenin pathway [65]. Based on these observations, we infer that *mrc2* may play an indirect role in the sexual regulation of *B. purificata*.

In addition, transcriptome analysis showed that *tbx1* and *rnf216* exhibited significantly higher expression in the testis than in the ovary, whereas *mrc2* was markedly upregulated in ovarian tissue (Table S7). Based on these results, we propose that *rnf216*, *tbx1* and *mrc2* may represent novel candidate genes involved in sex differentiation in *B. purificata*. Nevertheless, further experimental verifications are required to investigate their functions and regulatory mechanisms during gonadal differentiation and development in *B. purificata*, such as via gene-editing assays and comprehensive gene expression profiling of gonads across key sex determination and differentiation stages [62].

*Mis18bp1*, which encodes MIS18 bonding protein 1 (MIS18BP1), is a core subunit of the MIS18 complex (MIS18*α*/MIS18*β*/MIS18BP1). In the present study, numerous SNPs and InDels were detected within the *mis18bp1* locus, and transcriptome profiling showed that this gene was significantly upregulated in the testis compared with the ovary. However, no direct evidence currently supports the involvement of *mis18bp1* in sex differentiation or gonadal development. Previous studies have documented that *mis18bp1* exerts essential functions in mitotic progression and kinetochore protein assembly in eukaryotes, interacts with diverse protein partners, and primarily participates in mitosis and cell cycle regulation [66]. In addition, genes closely related to *mis18bp1* are predominantly implicated in biological processes such as the cell cycle, chromosome segregation, and DNA repair [66]. On this basis, we hypothesize that *mis18bp1* may contribute indirectly to sexual regulation in *B. purificata*. Further in-depth functional studies will be necessary to elucidate the precise role of *mis18bp1* during sex differentiation and gonadal development in this species.

Accumulating evidence indicates that sex determination in freshwater snails is primarily governed by genetic factors, and to date, no environmental factors have been identified that influence their sex ratio. For instance, the sex of the apple snail *Pomacea canaliculata* is controlled by a limited set of nuclear sex-determining genes, rather than cytoplasmic factors (such as mtDNA) [67,68,69]. The genetic basis of sex determination in viviparid snails has also been supported by analyses of family sex ratios and karyotypes [70]. Karyotype investigations suggest that certain species (e.g., *Viviparus ater*, *V. mamillatus* and *V. acerosus*) possess a ZW/ZZ sex-determining system [71], while an XX/XY pattern has been documented in other species, including *Viviparus subpurpureus* and *Tulotoma angulata* [72,73]. In the present study, we found that *B. purificata*, a gonochoric species, may primarily follow an XX/XY sex-determination pattern. This speculation is based on the segregation patterns of sex-specific SNPs and InDels. For example, of the 571 significant sex-differentiated SNP loci, females were predominantly homozygous, whereas males were mostly heterozygous. Furthermore, the sex-specific InDel marker produced only one 82 bp band in females, whereas two bands (73 and 82 bp) were present in males on agarose electrophoresis gel, which is a typical pattern for an XX/XY sex-determining system. Nevertheless, it should be noted that minor genes potentially involved in sex determination may exist on other chromosomes. The main results obtained here were consistent with previous findings in closely related species [70]. These results will be helpful for further exploration of the sex-determination mechanism of *B. purificata*.

**Figure 8:**
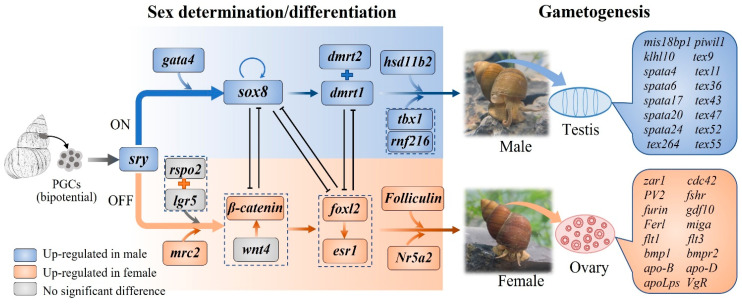
Predicted regulatory network and key candidate genes associated with sex determination, differentiation, and gonadal development in *B. purificata*.

Integrating the DEGs from RNA-Seq and candidate genes from GWAS, we hypothesized the possible regulatory network of sex determination, differentiation, and gonadal development in *B. purificata*, as shown in Figure 8. *Sry* may act as a key regulator in the sex-determining pathway of *B. purificata*, given that it functions as the master switch for testicular development in vertebrates and exhibits strict testis-specific expression. *Sry* directly activates the expression of *sox8*, which has been proven to be an alternate testis-differentiating factor of *sox9* in mice [74]. The *sry*/*sox8* suppresses ovarian-promoting genes and activates many male-specific genes, such as *dmrt1* and *dmrt2*, leading to the differentiation of Sertoli cells [75]. In the absence of *sry*, the Wnt/β-catenin signaling pathway was activated by *rspo2*/*igr5* and *mrc2*, which suppresses *sox8* expression, allowing granulosa cell differentiation, and in turn, ovarian differentiation. In addition, *foxl2* and estrogen receptor (*esr1*) expression are required to actively repress *sox8* and *dmrt1*/*2* expression to maintain ovarian development [76]. Following gonadal differentiation, the distinct types and expression levels of hormones secreted by the testes (e.g., *hsd11b2*) and ovaries (e.g., *folliculin*) determine the development of most secondary sexual characteristics. Our findings, together with previous reports [77,78], suggest that sex determination and differentiation in *B. purificata* may share more similarities with those in other vertebrates than with those of teleost fishes. The working model and novel insights obtained in this study are expected to promote further research into the sex-determining pathways in freshwater gastropods and other mollusks.

## 5. Conclusions

Our results indicate that *B. purificata* potentially follows a primarily XX/XY sex-determination system, with *sry*, *sox8*, *dmrt1* and *dmrt2* being critical in male sex differentiation, while *β-catenin*, *foxl2*, *esr1*, and *nr5a2* play important roles in female sex differentiation. In addition, *mis18bp1*, *rnf216*, *tbx1*, and *mrc2* may be important sex-associated candidate genes. However, a noticeable constraint in this study is that our transcriptomic profiles were only obtained from mature gonads, which may result in the exclusion of genes functioning during early sex determination. A sex-linked InDel marker was successfully developed, which can distinguish males and females cost-effectively. Nevertheless, population-specific variation may exist in some populations. Future investigations should prioritize early gonadal stages, broader sampling, and validation, as well as functional validation via gene editing and expression profiling. Together, these results provide a useful foundation for elucidating the genetic mechanism of sex determination and for developing monosex stocks in *B. purificata*.

## Figures and Tables

**Table 1:** Representative DEGs implicated in sex determination, differentiation, and gonadal development within the *B. purificata* transcriptome.

	Gene ID	Annotation	Gene Symbol	Log_2_FC (♂/♀)	Sex Bias
Sex determination/differentiation	Bpur00001-RDQH	Sex-determining region Y protein	*sry*	8.94	testis
	Bpur02157-RA	Transcription factor GATA-4	*gata4*	7.31	testis
	Bpur23567-RA	Doublesex- and mab-3-related transcription factor 1	*dmrt1*	4.55	testis
	Bpur00001-RDF	Doublesex- and mab-3-related transcription factor 2	*dmrt2*	2.89	testis
	Bpur22874-RA	Transcription factor sox-8	*sox8*	2.75	testis
	Bpur11206-RA	GATA zinc finger domain-containing protein 1	*gatad1*	2.30	testis
	Bpur12501-RA	Transformer-2 protein homolog beta	*tra2β*	1.50	testis
	Bpur00001-RLO	Structural maintenance of chromosomes protein 1A	*smc1a*	1.37	testis
	Bpur07492-RA	Protein dpy-30	*dpy-30*	3.39	testis
	Bpur09837-RA	Prolyl 4-hydroxylase subunit alpha-1	*dpy-18*	11.22	testis
	Bpur23672-RA	Catenin beta	*β-catenin*	−1.84	ovary
	Bpur06339-RA	Estrogen receptor	*esr1*	−1.27	ovary
	Bpur14674-RA	Nuclear receptor subfamily 5 group A member 2	*Nr5a2*	−3.03	ovary
	Bpur25373-RA	Forkhead box protein L2	*foxl2*	−1.48	ovary
	Bpur13364-RA	Structural maintenance of chromosomes protein 3	*smc3*	−2.60	ovary
	Bpur12022-RA	C-type mannose receptor 2	*mrc2*	−3.52	ovary
Ovarian development	Bpur13901-RA	Zygote arrest protein 1	*zar1*	−2.19	ovary
	Bpur20846-RA	perivitellin-2	*PV2*	−6.43	ovary
	Bpur14638-RA	Vitellogenin receptor	*VgR*	−0.31	ovary
	Bpur13362-RA	Yolk ferritin	*ferl*	−6.84	ovary
	Bpur11501-RA	Bone morphogenetic protein 1	*bmp1*	−1.83	ovary
	Bpur11216-RA	Bone morphogenetic protein receptor type-2	*bmpr2*	−7.29	ovary
	Bpur00001-RBE	Apolipophorins	*apoLps*	−1.77	ovary
	Bpur00643-RA	Apolipoprotein B	*apo-B*	−1.34	ovary
	Bpur00001-RDJD	Apolipoprotein D	*apo-D*	−4.91	ovary
	Bpur06429-RA	Follicle-stimulating hormone receptor	*fshr*	−1.38	ovary
	Bpur22319-RA	Growth/differentiation factor 10	*gdf10*	−3.09	ovary
	Bpur14579-RA	Mitoguardin	*miga*	−2.14	ovary
	Bpur09879-RA	Cdc42	*cdc42*	1.55	testis
	Bpur04778-RA	Furin	*furin*	−2.27	ovary
	Bpur18860-RA	Vascular endothelial growth factor receptor 1	*flt1*	−10.79	ovary
	Bpur01752-RA	Vascular endothelial growth factor receptor 4	*flt4*	−2.21	ovary
Spermatogenesis	Bpur27519-RA	Piwi-like protein 1	*piwil1*	2.51	testis
	Bpur20423-RA	Spermatogenesis-associated protein 4	*spata4*	4.74	testis
	Bpur00486-RA	Spermatogenesis associated 6	*spata6*	4.25	testis
	Bpur07203-RA	Spermatogenesis-associated protein 17	*spata17*	2.67	testis
	Bpur22988-RA	Spermatogenesis-associated protein 20	*spata20*	2.06	testis
	Bpur12601-RA	Spermatogenesis-associated protein 24	*spata24*	2.28	testis
	Bpur13372-RA	Kelch-like protein 10	*klhl10*	10.64	testis
	Bpur19434-RA	Testis-expressed protein 9	*tex9*	1.87	testis
	Bpur16358-RA	Testis-expressed protein 11	*tex11*	8.25	testis
	Bpur12360-RA	Testis-expressed protein 36	*tex36*	2.09	testis
	Bpur06417-RA	testis-expressed protein 43	*tex43*	4.45	testis
	Bpur00001-RFBM	Testis-expressed protein 47	*tex47*	5.28	testis
	Bpur00001-RBTA	Testis-expressed protein 49	*tex49*	3.18	testis
	Bpur00001-RFQR	Testis-expressed protein 52	*tex52*	5.99	testis
	Bpur05705-RA	Testis-specific expressed protein 55	*tex55*	3.62	testis
	Bpur00001-RWC	Testis-expressed protein 264	*tex264*	1.70	testis
	Bpur00001-REWA	Meiotic recombination protein DMC1/LIM15 homolog	*dmc1*	2.98	testis
	Bpur00745-RA	Meiotic recombination protein SPO11	*spo11*	5.29	testis
	Bpur25494-RA	Ropporin-1-like protein	*ropn1l*	6.52	testis
	Bpur00001-RCNY	Sperm flagellar protein 1	*spef1*	4.24	testis
	Bpur01300-RA	Sperm flagellar protein 2	*spef2*	4.00	testis
	Bpur22818-RA	Sperm-tail PG-rich repeat-containing protein 2	*stpg2*	4.63	testis
	Bpur00001-REWW	Testis-specific serine/threonine-protein kinase 1	*tssk1b*	10.36	testis
	Bpur07524-RA	Testis-specific serine/threonine-protein kinase 2	*tssk2*	9.78	testis
	Bpur19652-RA	Testis-specific serine/threonine-protein kinase 4	*tssk4*	10.44	testis
	Bpur20343-RA	Motile sperm domain-containing protein 2	*mospd2*	1.84	testis
	Bpur14619-RA	Axonemal dynein light chain domain-containing protein 1	*axdnd1*	4.16	testis
	Bpur00001-RCGW	Sperm-associated antigen 6	*spag6*	3.64	testis
	Bpur05552-RA	G2/mitotic-specific cyclin-B3	*cycB3*	5.80	testis
Steroid biosynthesis/metabolism	Bpur09123-RA	11-beta-hydroxysteroid dehydrogenase type 2	*hsd11b2*	1.61	testis
	Bpur06879-RA	Cholesterol side-chain cleavage enzyme	*cyp11a1*	2.14	testis
	Bpur25569-RA	17-beta-hydroxysteroid dehydrogenase type 12	*17β-hsd12*	4.30	testis
	Bpur22604-RA	Folliculin	*FLCN*	−3.12	ovary
	Bpur18328-RA	Steroid 17-alpha-hydroxylase/17,20 lyase	*cyp17a1*	−2.11	ovary
	Bpur13151-RA	17-beta-hydroxysteroid dehydrogenase type 6	*hsd17b6*	−2.83	ovary
	Bpur01123-RA	Hydroxysteroid dehydrogenase-like protein 2	*hsdl2*	−1.32	ovary

**Table 2:** Sex-specific candidate genes overlapping between GWAS and RNA-Seq in *B. purificata*.

Gene ID	Gene Symbol	Gene Full Name	Chromosome No.	Location
Bpur04866-RA	*mis18bp1*	Mis18-binding protein 1	Chr. 2	34756547–34795747
Bpur23546-RA	*rnf216*	E3 ubiquitin-protein ligase RNF216	Chr. 5	48420400–48461075
Bpur00001-REEO	*tbx1*	T-box transcription factor	Chr. 3	13651545–13671647
Bpur12022-RA	*mrc2*	C-type mannose receptor 2	Chr. 1	132261558–132295171
Bpur11324-RA	*cubn*	Cubilin	Chr. 1	107556772–107648637
Bpur22275-RA	*sirt4*	NAD-dependent protein lipoamidase sirtuin-4, mitochondrial	Chr. 3	94100563–94107286
Bpur24902-RA	*dyps*	Dihydropyrimidinase	Chr. 4	4336227–4372805

## Data Availability

The whole-genome resequencing and RNA-Seq data are deposited in the NCBI BioProject database under accession number PRJNA1405152. Other datasets employed and analyzed in this study are obtainable from the corresponding author upon reasonable request.

## Supplementary Materials

The following supporting information can be downloaded at https://www.mdpi.com/article/10.3390/ani16060916/s1. Table S1: Sampling information of *B. purificata* in this study; Table S2: qRT-PCR primer information; Table S3: Sex-specific SNPs identified by GWAS; Table S4: Sex-specific InDels identified by GWAS; Table S5: Genotype of the sex-specific SNPs; Table S6: Sex-specific candidate genes obtained by GWAS; Table S7: Differentially expressed genes of testis and ovary; Figure S1: Expression patterns of 18 DEGs revealed by RNA seq and qRT PCR.
