# Supplementary material for: Elucidation of the XX/XY Sex Determination System and Development of a Sex-Linked Molecular Marker in the Freshwater Snail Bellamya purificata
Source: Animals (Basel). 2026 Mar 14;16(6):916. doi: 10.3390/ani16060916 (PMC13023353; doi:10.3390/ani16060916)
Supplement: Supplementary file 1 [file animals-16-00916-s001.zip › Table S1. Sampling information of B. purificata in this study.pdf]

Table S1. Sampling information of *B. purificata* in this study

Population	Location	Habitat	Sampling time	Sample No.	Male No.	Female No.	Body width (Range) (mm)	Body width (Mean±S.D.) (mm)	Body weight (Range) (g)	Body weight (Mean±S.D.) (g)
Qianjiang, Hubei Province	112°54′ E, 30°24′ N	Rice-crayfish coculture field	June and August, 2023	209	69	140	14.86~21.40	18.40±1.23	2.75~8.50	5.14±1.03
Chaohu Lake, Anhui Province	117°48′ E 31°36′ N	Lake	June and August, 2023	78	3	75	15.26~20.54	17.99±1.16	3.02~7.66	4.97±1.05
Honghu Lake, Hubei Province	113°24′ E, 29°48′ N	Lake	May 2024	150	53	97	14.40~20.83	18.13±1.09	3.01~8.37	5.08±0.96
Tianmen, Hubei Province	113°10′ E, 30°40′ N	Rice-crayfish coculture field	May 2024	138	66	72	14.73~20.35	18.25±1.17	3.12~7.38	5.03±0.95
Weishan Lake, Shandong Province	117°11′ E, 34°38′ N	Lake	May 2024	87	32	65	14.13~19.12	17.34±1.02	2.77~6.96	4.68±0.83
