# Supplementary material for: Elucidation of the XX/XY Sex Determination System and Development of a Sex-Linked Molecular Marker in the Freshwater Snail Bellamya purificata
Source: Animals (Basel). 2026 Mar 14;16(6):916. doi: 10.3390/ani16060916 (PMC13023353; doi:10.3390/ani16060916)
Supplement: Supplementary file 1 [file animals-16-00916-s001.zip › Table S2. qRT-PCR primer information.pdf]

Table S2. qRT-PCR primer information

Gene Name	Forward Primer Sequence (5′-3′)	Reverse Primer Sequence (5′-3′)	Product Size (bp)
sry	ACGGCAAAGAATGGTGGACA	ACGCTTGGGTTCATCAGACT	117
gata4	ATAGGCACACCAGCCTTTCC	TCTCTCGACCAACTTTCGCC	101
sox8	GGAACCCCTGGATGACCAAG	CTGTTGACCGTTCCTGACGA	261
dmrt1	CCCTTATGTCCGATGTGCGT	GGTCACTGGCTGTCATAGG	197
dmrt2	CGGTGTGGTGTCGTGTCTAA	CTTGAGCAGCCATGATTCGC	110
rnf216	GTGCCTCAACCCAGAGTGTT	GCTGCCGACAAACATAGCAA	245
β-catenin	GACGTGGAGACTATCTTGGCA	GCCTTGTCGGTGGTGAGAAA	155
nr5a2	CGTGCCATGAAGCAACAACA	TGTGGGAGAATTGGAGTGCC	183
rspo2	GGTGGTAACGCTTGTCCAGA	GTCTTTCCCTTCTCCCAGCC	236
foxl2	TGGCAATTAAGGAGTCGGGC	TGGAAACGGACACCCCAATG	238
spata6	CTGTAGCCTTTCCGGGCATA	CAAATGGAGGTCGAGGGTCA	231
klhl10	CCTACCTCCAACACGCCAAT	CAGTTCGTCAGCAGAGAGCA	269
tex11	GCAAGCAATGATGTCCAGCC	CATGGTAAGCTGCACAAGGC	205
Tssk2	ACTCGGGATGGCTGAAGATG	ACGAGACGACGAAGAACGAC	106
PV2	TGTTGTCTGGGGAGTTTCCG	ACTTGGTTCAGCCGACCTTC	111
bmp1	CTGCATACGGTGTTCTCGGT	TCAGACGGGTAGTTAGGCGA	163
VgR	GTGTCCGAGAAAATGCGTGT	TTCCTTCACAAGCTGCCTCG	132
fshr	GGGAGTGGTTTGCATGAGGA	CAACGTATTCACTGGCGCTG	106
ef1a	GCTCGACAGCTACCAAGGAC	GTTTGCTTGTGGAGTGTGGC	174
