# Supplementary figures and images for: Elucidation of the XX/XY Sex Determination System and Development of a Sex-Linked Molecular Marker in the Freshwater Snail Bellamya purificata
Source: Animals (Basel). 2026 Mar 14;16(6):916. doi: 10.3390/ani16060916 (PMC13023353; doi:10.3390/ani16060916)

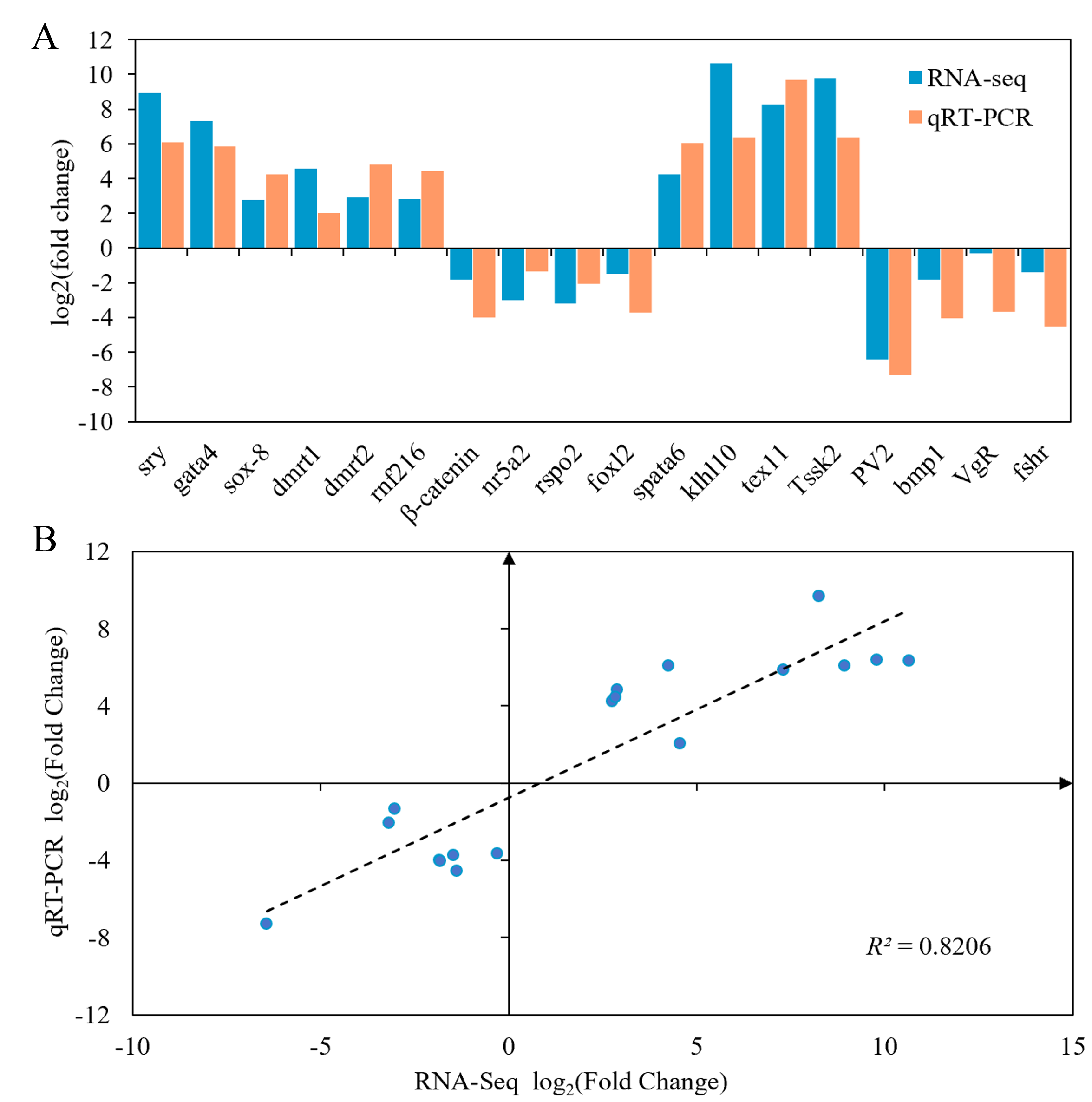

Supplement: Supplementary file 1 [file animals-16-00916-s001.zip › Figure S1. Expression patterns of 18 DEGs revealed by RNA seq and qRT PCR.tif]
